# Histological Comparison of Testicular Needle Biopsy and En Bloc Samples in Abattoir Calves

**DOI:** 10.3390/ani10050918

**Published:** 2020-05-25

**Authors:** Maike Rohländer, Henning Otzen, Kristina Rode, Klaus Jung, Marion Schmicke, Teresa Harborth, Marion Langeheine, Ralph Brehm, Árpád Csaba Bajcsy

**Affiliations:** 1 Clinic for Cattle, University of Veterinary Medicine Hannover, Foundation, Bischofsholer Damm 15, 30173 Hannover, Germany; teresa.harborth@tiho-hannover.de (T.H.); csaba.bajcsy@tiho-hannover.de (Á.C.B.); 2 ET-Service Schleswig-Holstein GbR, Jägerberg 1, 24960 Glücksburg, Germany; henningotzen@gmx.de; 3 Institute of Anatomy, University of Veterinary Medicine Hannover, Foundation, Bischofsholer Damm 15, 30173 Hannover, Germany; kristina.rode@tiho-hannover.de (K.R.); marion.langeheine@tiho-hannover.de (M.L.); ralph.brehm@tiho-hannover.de (R.B.); 4 Institute for Animal Breeding and Genetics, University of Veterinary Medicine Hannover, Foundation, Bünteweg 17p, 30559 Hannover, Germany; klaus.jung@tiho-hannover.de; 5 Institute for Agricultural and Nutritional Sciences, Animal Health Management, Martin Luther University Halle-Wittenberg, Theodor-Lieser-Straße 11, 06120 Halle (Saale), Germany; marion.schmicke@landw.uni-halle.de

**Keywords:** testis biopsy, histology, cattle, bull calf

## Abstract

**Simple Summary:**

The histological examination of testicular tissue offers a suitable diagnostic method for investigating possible testicular causes of reduced fertility. A diagnostic measure of testicular health or function that does not impair fertility is needed. One possibility could be a needle biopsy of the testis. The testicular biopsy provides the attending veterinarian with a useful tool for plastic evidence of testicular infertility causes. Since the representativeness of this method in equine medicine was questioned, and has not been sufficiently answered in young bull calves so far, this study was carried out on male calves that were sent for slaughter. The aim of this study was to evaluate the significance of needle biopsy compared to en bloc resection. No differences between the individual locations of the tissue samples could be found. The results of the needle biopsy differ significantly from those of the tissue samples in almost all locations. This suggests that needle biopsy does not provide the same histological information as the tissue samples to determine the fertility status of the testes of bull calves.

**Abstract:**

The aim of this study was to test whether a single testicular needle biopsy could provide histological results comparable to en bloc resection histology and whether one biopsy was sufficient to reflect the histology of an entire pair of testicles. Two methods of sample collection were tested on 32 bull calves aged five to eight months to compare histological parameters of needle biopsy with those of en bloc resection samples. One testicular needle biopsy of the right and three en bloc samples of both testicles were collected and compared for the number of tubular cross sections, tubules with elongated spermatids (ES), outer/inner diameter of tubules, thickness of tubular wall, and number of Sertoli cells (SC). Additionally, animal data were considered. No significant differences were found between the left and right testis or among the individual locations of en bloc samples. However, histologically significant differences (Bonferroni-adjusted significance level: *p <* 0.05/6 = 0.0083) were found between the needle biopsy and en bloc resection regarding the tubular cross sections per visual field (*p <* 0.05), the outer (*p =* 0.01) and inner diameter and the thickness of the tubular wall (both *p <* 0.01). In the SOX9 immunohistochemical staining, no significant differences (*p >* 0.05) could be observed for SC numbers between needle biopsy and en bloc samples. In conclusion, results of testicular needle biopsy do not have the same validity as the en bloc resection histology. Furthermore, one biopsy is insufficient to reflect the histology of the entire testicular pair.

## 1. Introduction

Needle biopsy has been advocated as an instrument for fertility status examination in different species [1,2]. As the effects of the biopsy on patients are discussed elsewhere (men: [3]; stallions: [4]; bulls: [2,5]), this study focused on the histological comparability of needle biopsy samples versus en bloc samples as well as on the validity of a single biopsy for both whole, physiologically developed testes.

Fertility evaluation in bulls has always been a great challenge. Various methods exist, all of them examining a certain aspect of fertility, e.g., semen evaluation or gene tests [6,7]. Yet, not all causes of in- or subfertility can be sufficiently explained. This study investigated the method of testicular needle biopsy, which could provide a detailed histological evaluation of coherent testicular tissue, potentially in-vivo. Requiring only a minor intervention, just slightly more invasive than a fine-needle aspiration biopsy but recovering a coherent tissue sample similar to an en bloc resection, needle biopsy seemed to combine the advantages of the two methods regarding ease of use and analyzability.

Previous studies have shown some applications for needle biopsy, e.g., in humans [8] or stallions [4]. However, to our knowledge, there are no examinations in which testicular biopsy is evaluated based on en bloc resection.

Histologically, the testis is composed of the intertubular part consisting of the vascular connective tissue apparatus, the Leydig cells, and the tubular component. This distribution changes with increasing age in favor of the tubules, so that between the ages of seven and twelve months, the tubules already constitute 80% of the testicular volume. In adult bulls, the intertubular compartment accounts for only 15% [9].

The S-shaped course of testicular growth first increases after about six months and continues through puberty, slowing down again when the sperm production of an adult animal is reached [10,11]. Following testicular growth, the tubular diameter gradually expands until it increases even faster after five to six months [10,11]. At the same time, seminiferous tubules continue to grow in length and lumen formation takes place. Hereby, the tubular diameter is determined [12]. Responsible for the testicular growth of the first eight months is the increase in tubular diameter and length as well as number of germ cells [13]. The immature SC population multiplies between the first and about the fifth month postnatally and differentiates further into mature SC. The final SC differentiation seems to be finished between 7.5 and ten months [10,11,14]. The seminiferous cord shows gonocytes around parturition, which continue to develop after about 7.5 months [13,14]. First spermatocytes appear between five- and 7.5-months of age, while round and elongated spermatids start to differentiate between six and nine months [10,11,14]. Between the ages of eight and ten months, mature spermatozoa can be found [10,11,12].

In order to test the histological validity of needle biopsy, a study population with potential differences in testicular histology and between animals and within testes was required. As already mentioned, sub- and infertility can have many causes and a study population of such impaired, comparable bulls was not at our disposal; thus, young bull calves going for slaughter were used as a first study population. The beginning of puberty is determined from about eight months onwards in various cattle breeds [15,16], so that the greatest changes in growth can be expected in the preceding months.

The aim of this study was, therefore, to test whether a single testicular needle biopsy could provide histological results comparable to en bloc resection histology and whether one biopsy was sufficient to reflect the histology of an entire pair of testicles.

## 2. Materials and Methods 

### 2.1. Animals

Data and samples of intact male calves (n = 32) were collected immediately after slaughtering at a regular abattoir. Five animals were represented in the five-month-old, ten each in the six- and seven-month-old, and seven calves in the eight-month-old age group (Table 1). The mean age ± SD was 6.6 ± 1.0 months (min = five months, max = eight months).

### 2.2. Collection of Testicular Samples

Testicular samples of the 32 male calves were collected on four different dates between the end of April and the end of June 2018 in an abattoir in Mecklenburg-Western Pomerania, Germany.

All samples were collected after the slaughter process and subsequently numbered in order to be able to assign each sample to the corresponding animal. Furthermore, photographs were taken during the processing.

Immediately after the testicles within the scrotum of the calves had been removed at the slaughter line by the abattoir-staff, they were placed on freezer packs. These organs were then prepared for further analyses within seven consecutive hours on-site.

The testicular pairs were prepared by first cutting the scrotum medially and then uncovering the right and then the left testicle within the tunica vaginalis. Attention was paid to any existing alterations such as hematomas or adhesions, e.g., between the testicle and the tunica vaginalis. Afterwards, the testes were weighed individually.

**Figure 1:**
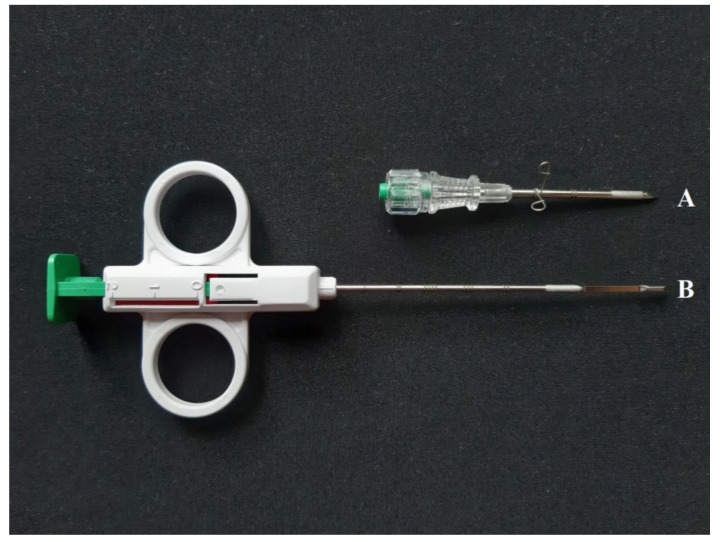
SuperCore ^TM^ Biopsy Instrument (B) (14 G × 9 cm) with Co-Axial Introducer Needle (**A**) (13 G × 3.9 cm). The biopsy needle has two tension points to pre-tension it, as the numbers 0, 1, and 2 illustrate. In the present study, tension was applied up to the second (2) tension point. At the tip of the biopsy needle (**B**) is the recess where the tissue sample can be found after the plunger has been pressed.

A single testicular needle biopsy was performed on the right testis using the SuperCore™ biopsy instrument (Argon Medical Devices Inc., Athens, TX, USA) (Figure 1). The tissue cylinder, which is obtained by punching out, has a size of 10 × 1.5 mm. The trocar sleeve (Figure 1A) was punctured from the lateral side at the transition from the proximal to the middle third of the right testis. The trocar sleeve was inserted at an acute angle in a distal direction. The biopsy needle (Figure 1B), which is a semi-automatic system with two tension points, was then inserted and the sample was taken from the distal portion of the testis. The biopsy needle was always pretensioned up to the second position so that a biopsy sample approximately 1 cm in length and 1.5 mm in width could be taken. The biopsy sample was immediately transferred into Bouin’s solution (10% formaldehyde, 4% picric acid, 5% glacial acetic acid) and allowed to diffuse for 24 hours.

Following the needle biopsy, both testes were dissected using the same procedure, starting with the right one. Each testis was divided into longitudinal halves, so the rete testis could be identified in the longitudinal axis of the testis. Next, en bloc samples of approximately 1 cm^3^ in size were taken from three different locations (proximal, middle, distal), as described by Brito et al. [17] and Da Silva [18]. The samples were placed into Bouin’s solution for 48 h.

### 2.3. Histological Processing

Histological processing followed a standardized procedure [19]. The Bouin-fixed samples were embedded in paraffin and cut into 3 µm-thick sections using a sledge microtome (Jung AG, Heidelberg, Germany). The sections were then stained with haematoxylin-eosin (HE) and evaluated with a light microscope (Axioskop, Carl Zeiss AG, Oberkochen, Germany) [4]. The used field of view of the microscope was 438 × 331 µm.

The following parameters were determined: number of tubular cross sections per visual field, number of tubules with ES, outer tubular diameter (i.e., extending up to the end of the tubular wall), inner tubular diameter (i.e., the lumen, extending up to the adluminal end of the germinal epithelium, with one contiguous center), thickness of the tubular wall, and number of SC per tubular cross section. Ten randomly selected visual fields were examined in each of the en bloc samples (n = 60 per animal). The number of tubular cross sections were counted per visual field using a magnification of 100 (objective: 10 ×, ocular: 10 ×), whereas the number of tubules with ES, the number of SC per tubular cross section as well as the outer and inner tubular diameters, and the thickness of the tubular wall were determined using a magnification of 200 (objective: 20 ×, ocular 10 ×). The same parameters were determined for the needle biopsy samples, but the entire tissue material was evaluated, which corresponds to approximately four visual fields. All sections were analyzed by the same person.

### 2.4. Immunohistochemistry

**Figure 2:**
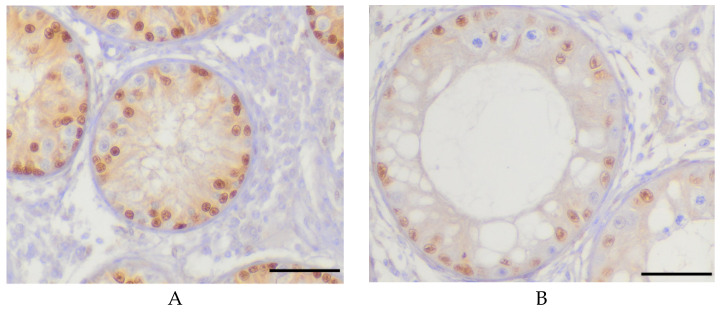
Representative immunohistochemical microphotographs of testicular tissue sections of en bloc samples from calves of the younger (**A**: five months old) and older (**B**: eight months old) age group. Scale bars = 50 µm. The images represent SOX9 stained samples. SOX9 staining shows the brown coloring of the Sertoli cells (SC) nuclei and germ cells are immune-negative (blue).

In order to verify the method of SC counting described above, immunohistochemistry (IHC) was performed on Bouin-fixed paraffin embedded sections. In mice and rats, it was described that SOX9 staining reliably stains the SC nuclei so that they contrast clearly with adjacent germ cells [20]. This characteristic was also observed in bulls [21] (Figure 2A,B).

The preparation was the same as for the HE sections. First, the paraffin was removed using xylene and the endogenous peroxidase activity was inhibited with 3% hydrogen peroxide (H_2_O_2_) in 80% ethanol for 30 min. The sections for SOX9 were pre-treated with sodium citrate buffer (pH 6.0) for 20 min on a heating plate at 96 to 99 °C. Afterwards, these were then cooled down at room temperature for 30 min, then blocked with 3% bovine serum albumin for 20 min and incubated overnight with the respective primary antibody (SOX9, 1:400, Millipore AB5535, Millipore Inc., Temecula, CA, USA). The preparation of the sections with the secondary antibody system, EnVision^TM^ + Kit HRP Rabbit DAB + (Dako Deutschland GmbH, Hamburg, Germany, catalogue number: K4011) took 45 min. After visualization with DAB, the sections were counterstained with haematoxylin for two seconds and then washed under running water for ten minutes. After dehydration, all slides were mounted with Eukitt^TM^ (O. Kindler GmbH, Freiburg, Germany).

### 2.5. Statistical Analysis

Statistical evaluation was carried out using SAS 9.4m5 with the Enterprise Guide Client 7.15 (SAS Institute Inc., Cary, NC, USA). The level of significance was set at alpha = 5%. Initially, the Shapiro–Wilk test was performed to test continuous variables for normal distribution. Variables that were found to be normally distributed were analyzed using the ANOVA. Specifically, ANOVA for repeated measurements was performed considering sample localization, side and ear tag number as independent variables, and histological data as dependent variables to detect significant differences between sample locations within a calf and testis. Furthermore, for non-normally distributed variables, Wilcoxon’s rank sum test was used specifically to compare the respective sample locations of the en bloc samples with the testicular needle biopsy. In addition, Pearson’s correlation coefficient r was used to study the correlation of age, carcass, and total testicular weights with the various macroscopic and histological data. For non-parametric tests and for calculating correlation coefficients, repeated measurements were averaged for each individual.

Ethic statement: no living animals were used in the present study. Complying with the German Animal Welfare Act, in accordance with section five, paragraph seven, section two, sentence three, this experiment was not an animal experiment.

## 3. Results

### 3.1. General Data of the Animals

The average carcass weight was 142 ± 33 kg (min = 54.5 kg, max = 220.5 kg).

The average total testicular weight was 187 ± 88 g (min = 66 g, max = 424 g), with the right testicle weighing 95 ± 45 g (min = 33 g, max = 210 g) and the left testicle 91 ± 43 g (min = 33 g, max = 218 g). The mean value comparison of the weights of the right and left testis resulted in a *p*-value of 0.73.

Within the abattoir data, carcass weight (*p =* 0.47) and total testicular weight (*p =* 0.35) were not significantly correlated with age, whereas a positive correlation between carcass weight (*p <* 0.01) and total testicular weight was found (r = 0.56).

### 3.2. Data of the Histological Examination

#### 3.2.1. Investigations in HE Staining

No significant differences (*p >* 0.05) were found between the right and left testicle for the evaluated histological parameters. After pooling the left and right testes for each site (proximal, middle, and distal), pairwise analysis between en bloc sample sites revealed only non-significant differences, indicating similar tissue architecture (Table 2).

**Figure 3:**
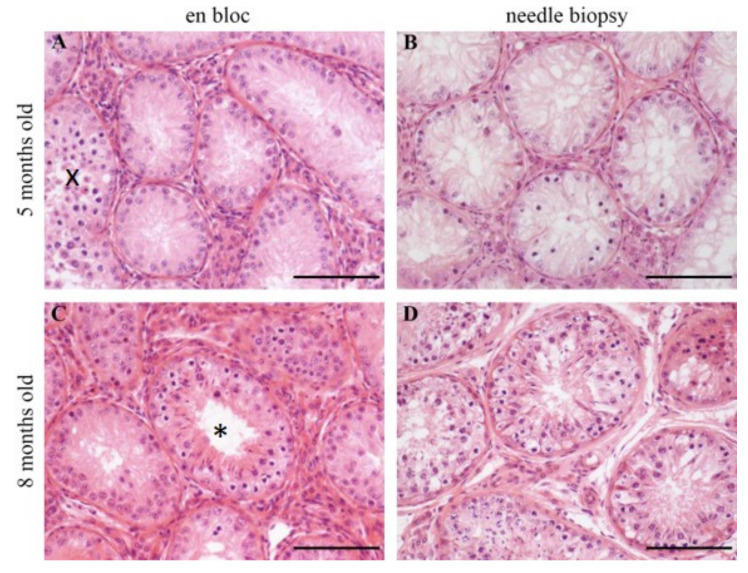
Representative haematoxylin-eosin (HE) microphotographs of testicular tissue sections of calves of the younger (five months old) and older (eight months old) age group. Scale bars = 100 µm. **A** and **C** represent en bloc samples and **B** and **D** represent needle biopsy samples. En bloc samples obtained conventionally (A, C), testicular morphology, e.g., tubular lumen (3C asterisk) are better preserved than needle biopsy samples (B, D). In younger bull calves (A, B), advanced spermatogenic cells (e.g., spermatocytes) appear rarely but are still visible compared to older animals (C, D). In younger calves (A), a tubule containing spermatocytes is present (x).

The histological comparison of en bloc and needle biopsy samples revealed that the mean morphology of the en bloc samples (Figure 3A,C) was clearly better preserved than that of the needle biopsy samples (Figure 3B,D). Particularly evident was the often markedly smaller or missing lumen (Figure 3C,D, asterisk), as well as the presence of irregular tubules in needle biopsy samples.

Histological evaluation of HE-stained samples revealed significant differences between en bloc and needle biopsy samples regarding the number of tubular cross sections, the number of SC per tubular cross section, the outer tubular diameter, the inner tubular diameter, and thickness of the tubular wall (Table 3). The percentage ratio of tubules with ES to tubule sections is 8.3% for en bloc samples and 7.2% for needle biopsy samples (*p >* 0.05).

Comparing histological evaluation results of the needle biopsy samples with corresponding values of en bloc sampling sites of the right testis revealed mean number of tubules with ES between the proximal en bloc and needle biopsy sample were similar (Table 4).

After dividing the samples according to age of the bull calves, tubules with ES were detected earlier in en bloc than in needle biopsy samples (Table 5).

When en bloc samples were grouped according to age (five to six months vs. seven to eight months), older animals had fewer tubules per visual field, but these were larger and contained more tubules with ES. The number of tubules with ES (r = 0.30), outer (r = 0.25) and inner tubular diameter (r = 0.42) and thickness of the tubular wall (r = 0.31) increased with age, whereas the number of tubular cross sections per visual field (r = −0.27) decreased with age (all with *p <* 0.001). Furthermore, the number of detected SC per tubular cross section differed significantly between the younger and older animals in HE-staining (*p =* 0.0033).

In the en bloc samples, statistically strong significant correlations (*p <* 0.001) were found between the carcass weight and the number of tubular cross sections (r = −0.49), the outer (r = 0.47) and inner tubular diameter (r = 0.26) and the thickness of the tubular wall (r = −0.26). With increasing carcass weight, the number of tubular cross sections and the amount of tubular wall thickness decreased, whereas the other parameters (outer/inner tubular diameter) increased. In addition, the total testicular weight had a positive effect on the number of tubules with ES (r = 0.30, *p <* 0.001).

The histological data of the needle biopsy specimens (excluding the outer tubular diameter *p =* 0.0022) were not significantly correlated with the carcass weight. Additionally, only the inner tubular diameter was significantly correlated with age (*p =* 0.0033).

#### 3.2.2. Investigations in Immunohistochemistry

When repeating the counting procedure using immunohistochemical SOX9 staining (Figure 2), however, no differences between en bloc and needle biopsy could be detected (Table 6).

## 4. Discussion

The aim of this study was to evaluate whether histological results were comparable between en bloc resection and needle biopsy of testicular tissue. Furthermore, it was examined whether one needle biopsy could be sufficient to reflect the histology of the entire testicles if they developed homogeneously and physiologically.

Needle biopsy was chosen because it combines the advantages of fine needle aspiration and open biopsy. The advantage of fine needle aspiration is that it has a lower complication rate [22] than open biopsy. In addition, no preceding sedation or anesthesia is required [23]. The major disadvantage is the necessity for an histopathological evaluation of the taken samples, which relies on few individual cells [3], the evaluation being dependent on the cytologist’s experience [8]. In the case of open biopsy, a greater continuous piece of tissue can be obtained, which makes microscopic evaluation a great deal easier [24]. Recovery time after the en bloc resection surgery is accompanied by a lengthy healing process or results in irreversible damage in live animals [25]. On the contrary, needle biopsy is minimally invasive. Nevertheless, a coherent tissue can be obtained for histological evaluation. This technique was reported to be safe and practicable in bulls [2] and stallions [4], as well as simple and cost-effective in humans [26]. However, a study verifying the needle biopsy with en bloc biopsy has been missing to date.

In the presented study, material originated from abattoir calves. The animals used here had an average slaughter weight of 142 ± 33 kg. Thus, the development of these five- to eight-month-old calves was in accordance with the studies by Bagu et al. [14], assuming a slaughter yield of 60 % [27]. The mean values of the individual testicular weights did not differ significantly between the right and the left testes, so that it was assumed that the postnatal and physical development of the testes did not differ.

Previous reports have shown that even in mature animals, no significant difference can be found between the two testicles regarding weight [28] or histological preparation (shrinkage, density) [29], as is the case in other species, i.e., in boars [30].

In the present study, both carcass weight (*p =* 0.47) and total testicular weight (*p =* 0.35) were not significantly correlated with age, which is surprising and contrary to previous investigations [31,32]. Nonetheless, this depends on different determinants. There were other factors influencing the results in the present study, for example, different breeds were present, animals had different farms of origin and were therefore kept and fed differently. Moreover, the sample size (n = 32) was too small to allow a valid statement regarding this aspect.

With increasing carcass weight, the bull calves had significantly heavier testicles (*p <* 0.01), which was consistent with the findings of Kastelic [32]. The comparison of the mean value of the en bloc samples of the different locations showed no significant relationships for the six different histological parameters according to the Bonferroni-corrected significance level (*p >* 0.0083, Table 2). Thus, it can be assumed that testicular parenchyma is homogeneous throughout the entire organ, which coincides with findings of other researchers [28,29,30]. Elsewhere, it has already been described in bulls [29,33] and other animal species (boars [30] and stallions [34]) that testicles develop uniformly and that a sample from a single region of the testicle is sufficient to make a valid statement about the condition of the testicle.

Histological evaluation of the en bloc and needle biopsy samples revealed that more artefacts like missing lumen were found in needle biopsy samples. Reason for these artefacts might be the tissue compression caused by the needle insertion. Another issue could have been the 24-hour fixation time in Bouin’s solution, which might have been too long for the small tissue pieces of the needle biopsy.

The poor agreement generated between en bloc and needle biopsy samples is indicative of the two methods delivering only limited comparable results and might have the same causes as the observed artefacts.

These two effects could explain why the number of SC per tubular cross section was different in needle biopsy samples depending on staining. The pressure exerted on the tissue by the biopsy needle could also affect the traceability of the SC, as only a small amount was found in HE-stained needle biopsy samples compared to en bloc samples. However, by employing an SOX9 staining it was possible to identify previously masked SC in HE, so that no differences in the SC count per tubule and between the five- and eight-month-old animals were evident anymore. Therefore, SOX9 staining should be used for SC identification in needle biopsy. This leads to the assumption that SC proliferation had already been completed in the five-month-old animals and that the SC count had already been determined by 20 weeks of age. This is partly supported by the findings of Bagu et al. [35] and Barth et al. [31], who observed that the final SC count is already determined during calfhood because SC proliferation ends at 20 to 25 weeks. In the present study, SC proliferation was presumably already completed at the age of 20 weeks, i.e., right at the beginning of the above-mentioned period.

The histological difference between the number of tubules with ES in en bloc and needle biopsy could have profound effects. Seminiferous tubules with ES were found in en bloc samples of every age group and more frequently. In needle biopsy samples, no tubules with ES were found in the five-month-old calves. Since the existence of these spermatids is a necessary step in the production process of potentially fertile ejaculates [11,13], the flawless identification would be an absolute requirement in determining, for example, the onset of productive spermatogenesis. The data of this study showed a tendency; further investigations with a larger sample per age group would be necessary to enable statistically validated statements.

En bloc samples showed a statistically highly significant negative relationship (*p <* 0.001) between age and number of tubular cross sections, meaning that as expected, the outer/inner tubular diameter and thickness of the tubular wall of seminiferous tubules enlarged with increasing age (each *p <* 0.001), resulting in fewer tubular cross sections per visual field. In addition, with increasing age, a lumen was formed that was not yet present or not as greatly pronounced in the five-month-old animals, which is in line with the findings of Evans et al. [12]. They concluded that the lumen formation takes place after six months. Other researchers also found that in the case of older animals, the ratio of testicular parenchyma to interstitial tissue increased [13,36]. This increase is related to tubular diameter, as in the present study, and length, both of which increase with age [13]. In addition, these changes are directly related to the number of SC and germ cells [31]. The number of SC is an important criterion for testicular size as each SC can only nourish a certain number of germ cells [31]. Berndtson et al. [33] also concluded that numerous SC and germ cells are associated with a higher testicular weight. Furthermore, it is known that the final number of SC is already determined during calfhood, since SC proliferation decreases and ends at the age of 20 to 25 weeks of age [31,35]. This is followed by the period of rapid testicular growth, which occurs after 25 weeks in the pubertal period [11]. The focus in this study was on the comparison of both methods, for which the evaluation of germ cells was not intended. For this purpose, an increased risk of false positive results was accepted in order to ensure that these results are reliable.

## 5. Conclusions

Needle biopsy does not have the same histological significance as en bloc specimens. In addition, histological differences can be observed compared to en bloc samples. Thus, the present study indicates that a single testicular needle biopsy is not suitable for assessing testicular tissue in comparison to en bloc samples. In a subsequent study, a larger sample size as well as more needle biopsy samples per testis should be evaluated. Furthermore, more effort needs to be invested in minimizing histological artefacts (such as lumen compression and fixation time), if needle biopsy should be considered as a useful, predictive diagnostic tool.

## Figures and Tables

**Table 1:** Number of animals (n = 32) per age group (months).

**Age (mo)**	5	6	7	8
**Number (n)**	5	10	10	7

**Table 2:** Test results (i.e., *p-*values) of the ANOVA (mixed model) comparing the three tissue sample locations among each other measured in haematoxylin-eosin (HE) staining. P-values were compared to the Bonferroni-adjusted significance level of alpha_Bonf_=0.0083, like in Table 4.

ANOVA – Mixed Model	Proximal – Middle	Proximal – Distal	Middle – Distal
Number of tubules with ES ^1^	0.11	0.01	0.67
Number of tubular cross sections	0.68	0.22	0.69
Outer tubular diameter	0.97	0.45	0.32
Inner tubular diameter	0.99	0.27	0.22
Thickness of the tubular wall	0.76	0.77	1.00
Number of SC ^2^ per tubular cross section	0.85	0.98	0.75

^1^ elongated spermatids; ² Sertoli cells

**Table 3:** Comparison of the mean values of the histological parameters of the en bloc samples (n = 192, proximal, middle distal) and the needle biopsy (n = 32) ± SD measured in HE staining.

Histological Parameter	En bloc Samples	Testicular NB ^3^	*p-*Value (Wilcoxon Test)
Number of tubular cross sections	241 ± 62	152 ± 63	<0.05
Number of tubules with ES ^1^	20 ± 28	11 ± 15	0.11
Outer tubular diameter	148 ± 27	134 ± 17	0.01
Inner tubular diameter	63 ± 31	29 ± 25	<0.01
Thickness of the tubular wall	1.87 ± 0.19	2.06 ± 0.24	<0.01
Number of SC ^2^ per tubular cross section	18 ± 5	14 ± 5	<0.01

^1^ elongated spermatids; ² Sertoli cells; ^3^ Needle biopsy

**Table 4:** Test results (i.e., p-values) of the ANOVA (mixed model) comparing the three different locations in en bloc samples (n = 192) with the needle biopsy (n = 32) measured in HE staining. In order to account for the multiple testing situation, P-values were compared to the Bonferroni-adjusted significance level of alpha_Bonf_ = 0.05/6 = 0.0083.

ANOVA—Mixed Model	Proximal—NB^3^	Middle—NB	Distal—NB
Number of tubules with ES ^1^	0.0170	0.0028	0.0028
Number of tubular cross sections	<0.0001	<0.0001	<0.0001
Outer tubular diameter	<0.0001	<0.0001	<0.0001
Inner tubular diameter	<0.0001	<0.0001	<0.0001
Thickness of the tubular wall	0.0012	0.0003	0.0002
Number of SC ^2^ per tubular cross section	0.0002	0.0002	<0.0001

^1^ elongated spermatids; ² Sertoli cells; ³ Needle biopsy

**Table 5:** Descriptive analysis of the distribution of the tubules with ES within the en bloc samples (n = 192) and within the needle biopsies (n = 32) as a proportion of the total number of animals per age group measured in HE staining.

Number of Animals With Tubules With ES ^1^ in the Different Sample Types		
Age (mo)	En bloc Samples	Needle Biopsy
5	1/5 (20%)	0/5 (0 %)
6	8/10 (80%)	7/10 (70%)
7	8/10 (80%)	6/10 (60%)
8	7/7 (100%)	7/7 (100%)

^1^ elongated spermatids

**Table 6:** Test results (i.e., *p*-value) of the mean value comparison of the SC count ± SD in en bloc and needle biopsy samples in SOX9-antibody or HE-staining. For SOX9 staining evaluating five young and two old bulls each per en bloc, all of the distal sample locations and the needle biopsy samples were used.

Staining	Mean Values of Number of SC ^1^ Per Tubular Cross Section		*p-*Value
	En Bloc Samples	Needle Biopsy Samples	
HE ²	17.9 ± 5.0	14.2 ± 4.9	0.0001
SOX9	21.5 ± 0.5	21.1 ± 1.0	0.4097

^1^ Sertoli cells; ² Haematoxylin-eosin
